# Motivations of Israeli physicians to return, or not, to Israel after their fellowship abroad

**DOI:** 10.1186/s13584-024-00652-6

**Published:** 2024-11-05

**Authors:** Lior Seluk, Daniel Weltsch, Gadi Segal, Mayan Gilboa

**Affiliations:** 1 Division of Pulmonary, Critical Care and Sleep Medicine, National Jewish Health, 1400 Jackson St, Denver, CO 80206 USA; 2 The Faculty of Medicine and Healthcare, Tel Aviv University, Tel Aviv, Israel; 3 Department of Orthopedic Surgery, Chaim Sheba Medical Center, Ramat-Gan, Israel; 4 Education Authority, Chaim Sheba Medical Center, Ramat-Gan, Israel; 5 The Infection Prevention & Control Unit, Sheba Medical Center, Ramat Gan, Israel

**Keywords:** Brain drain, Workforce migration, Professional mobility, Emigration patterns, Healthcare personnel shortage, Medical fellows

## Abstract

**Background:**

Emigration of healthcare professionals, particularly physicians, is an unresolved “pandemic”, influenced by various factors. In high-income countries, the training phase (fellowship) abroad is critical for career development, yet it poses challenges for the retention of these professionals upon their completion of training. This study aimed to identify the determinants influencing Israeli physicians' decisions to return to their home country after completing fellowship training abroad.

**Methods:**

This cross-sectional study conducted in early 2024, surveyed Israeli physicians with post-graduate training who pursued a fellowship abroad between 2013 and 2024. An anonymous survey included questions on demographics, training experience, and professional challenges. Analytical methods included descriptive statistics and regression analysis to explore factors associated with the decision to return.

**Results:**

Of the 323 individuals surveyed, 297 met the inclusion criteria. Among them, 141 (47.5%) have returned to Israel, while 156 (52.5%) currently reside abroad, 48 (31%) of them have completed their fellowship. Respondents identified several challenges, beginning with a complex application process prior to fellowship (47% stated this as a major challenge), substantial financial burdens averaging 35,546 USD in direct out-of-pocket expenses during, and job uncertainty, with only 55% having secured positions upon return. Of the 156 Israeli fellows still abroad, 107 (69%) reported medium to low confidence in returning. Factors associated with a higher likelihood of confidence in returning to Israel included a secured job position upon return (OR 8.6, 95% CI 3.1–28.9) and having an opportunity for a position that would utilize the skills gained during the fellowship (OR 3.5, 95% CI 1.3–10.2).

**Conclusion:**

The decision to return to Israel after a fellowship abroad is influenced by a mix of professional, personal, and geopolitical factors. To counteract the critical issue of brain drain, it is essential to enhance occupational certainty for returning physicians. These findings highlight the urgent need for healthcare policies that provide robust support for returning professionals and address their specific challenges.

**Supplementary Information:**

The online version contains supplementary material available at 10.1186/s13584-024-00652-6.

## Introduction

### Global trends in physicians’ emigration

Healthcare systems worldwide struggle with a significant workforce shortage, with an estimated global deficit of 5.9 million nurses and 4.3 million physicians [1]. Physicians, benefiting from universally applicable training, possess a high degree of mobility, enabling them to migrate with relative ease across international borders [2]. This phenomenon, known as “physician brain drain” [3, 4], significantly impacts not only low and medium-income countries [5] but also high-income nations. Countries like Ireland, the United Kingdom (UK), Greece, and Croatia are increasingly experiencing the emigration of local doctors and a growing dependence on immigrant doctors [2, 6–10]. Numerous studies have explored the factors driving doctors to leave their home countries [11, 12]; These can be broadly categorized into 'push' factors, which drive physicians away from their native countries, and ‘pull’ factors, which attract them to other nations [5]. These influences can be further broken down into macro (global and national), meso (professional), and micro (personal) levels [5, 13]. While specific geopolitical circumstances may vary, the underlying considerations for physicians are strikingly similar worldwide, especially among high-income nations [5, 10, 13–15].

### The situation in Israel

In high-income countries, including Israel, it is common for doctors to undertake a period of training abroad. This phase is pivotal for their career trajectory, and those who complete it often represent the elite in their field, and upon their return provide the substrate for the future professional leadership in their discipline [16].

The decision to return to their origin country after completing this phase is not obvious, and as new career opportunities are available in the new country, the health system in their origin country must consider what is the most effective way to “pull back” these physicians. It transcends mere migration, involving the intricate process of dismantling a life established in a foreign country to return to their homeland. It is imperative to comprehend the obstacles while enhancing the factors that can 'pull back' these internationally trained physicians, as this is crucial for healthcare systems aiming to sustain and cultivate future leadership. Effective utilization of resources providing essential support to these doctors may intensify their commitment to return and contribute to their original healthcare system post-fellowship.

### Aim of the current study

This study describes a survey focused on Israeli medical doctors who, after their post-graduate training in Israel, underwent additional training abroad, providing insights into the factors influencing their decision to return to their home country.

## Methods

### Study design and population

This cross-sectional study was conducted during the first quarter of 2024, to identify the determinants influencing the decision of Israeli physicians to return to their home country after completing fellowship training abroad. The study population consisted of Israeli physicians who had completed at least one residency in Israel and had undergone further medical training of at least six months duration outside of Israel post-2013. Official data on the number of Israeli doctors pursuing training abroad and their demographics does not exist. The life partners of participants who had already completed the survey were requested to exclude themselves to prevent bias.

### Survey development

The survey development process commenced with structured interviews (supplementary methods S1) with eight physicians including during fellowship, and post-fellowship, both among returnees and those who chose to remain abroad. These hour-long interviews, comprising 18 open-ended questions, were aimed at identifying key factors influencing the return decision and the challenges encountered. Based on the insights gathered, a preliminary survey was constructed and reviewed by 10 physicians who met the inclusion criteria, to assess the clarity, relevance, and comprehensibility of the questions. This feedback informed the final adjustments to the survey, resulting in a 72-question instrument. The survey, which lacked previously validated scales due to the specificity of the research topic, covered demographics, educational background, postgraduate training experiences, challenges, opportunities, financial implications, motives for staying abroad or returning, and preferences for future professional engagements. The survey created on the Microsoft Forms platform, aimed for a completion time of 15 min, and included a mix of single and multiple-choice questions, open-text responses, and Likert scale items to capture a broad range of data on demographics, professional experiences, and personal decisions regarding return versus (vs.) stay abroad (supplementary methods S2). The structured process described above was done with continuous consultation of a specialized epidemiologist and statistician.

### Survey dissemination

The dissemination strategy for the survey was designed to maximize reach and engagement among Israeli physicians training abroad, employing a multi-channel approach to ensure a comprehensive and diverse participant pool. The primary channels for survey distribution encompassed WhatsApp group chats, where the survey was shared in several pivotal WhatsApp groups tailored specifically to Israeli physicians abroad, spanning groups for those located in the United States of America (USA), Canada, Australia, the United Kingdom (UK), and Germany. Additionally, Facebook served as a platform for survey dissemination, with the survey being shared within prominent groups exclusively dedicated to Israeli physicians, characterized by significant membership and active engagement. LinkedIn played a role through personalized invitations, strategically targeting individuals identified within professional networks and associations. Moreover, direct emails were dispatched to members of several major Israeli medical associations, alongside proactive encouragement for participants to share the survey with other eligible physicians within their networks.

### Data analysis

Data analysis was performed using SPSS version 29 and R version 4.3.0. Descriptive statistics, including means, standard deviations, and proportions, were calculated. Inferential analyses involved t-tests for continuous variables and Pearson's chi-square or Fisher’s exact tests for categorical variables, depending on expected cell frequencies. The significance threshold was set at 0.05 for all tests.

A multivariable logistic regression analysis was limited to participants residing abroad, incorporating significant variables from univariate analyses (*p* ≤ 0.1) and intervention factors such as scholarships and guaranteed job opportunities Regression coefficients were exponentiated to obtain odds ratios. 95% confidence intervals for the odds ratios were calculated using the standard errors of the regression coefficients and were considered significant if not crossing 0. Missing data was handled by analyzing only complete cases. Since most questions were required, only 2 cases had missing data in the variables included in the regression analysis.

### Ethical considerations

The study protocol was approved by the Sheba Medical Center Institutional Review Board (IRB), (approval # SMC-1105–24). All participants provided informed consent before participation.

## Results

**Fig. 1:**
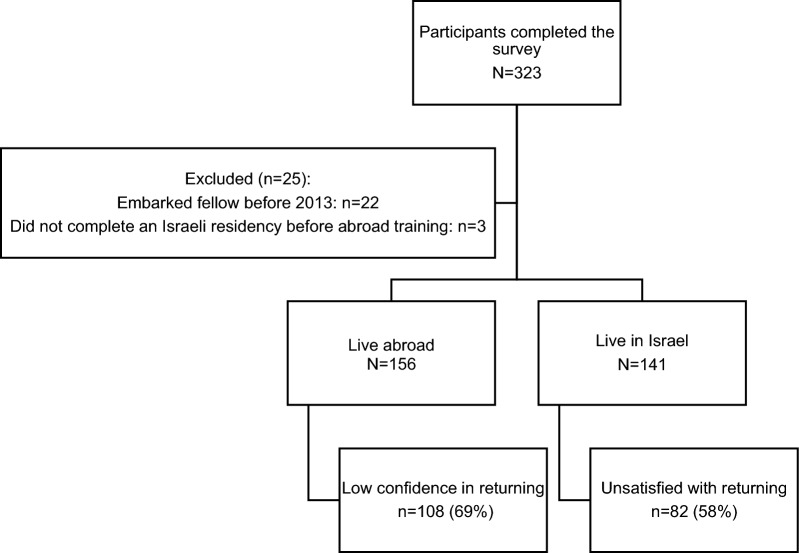
Flow chart of study participants

A total of 323 individuals responded to the survey. After applying inclusion criteria, 297 participants were eligible for the study. Of these, 156 are currently residing abroad, with 149 pursuing or having completed their fellowships in the same country. The remaining 141 have completed their fellowships and returned to their country of origin, Israel (Fig. 1).

### Demographics

The average age at the commencement of the fellowship was 38.4 (IQR 37–40). Females constituted 41% of the respondents. The average duration of the fellowship was 24.2 months. A significant majority (93.3%) embarked on their fellowships accompanied by a spouse and/or children, with an average of 2.4 (IQR 2–3) children per family, the average age range of the children varied between 3.3 (IQR 2–5) years for the youngest and 7.8 (IQR 5–10) years for the oldest. Detailed demographic characteristics, including medical specialties (a detailed list of medical specialties is presented in Supplementary Table S1) and countries of fellowships, are presented in Table 1.

**Table 1:** Demographic and professional characteristics of the 297 study participants

Variable	Category	N	%
Current place of residence	Israel	141	47.5
	Country of fellowship	149	50.2
	Other Country	7	2.4
Age—Mean (IQR)		38.4	(37–40)
Gender	Female	121	40.7
	Male	174	58.6
First year of fellowship	2022–2024	118	39.7
	2019–2021	102	34.3
	2016–2018	48	16.2
	2013–2015	29	9.8
Birth Country	Israel	255	85.9
	Non-Israel	42	14.1
Ethnicity	Jewish	275	92.6
	Non-Jewish	20	6.7
Place of residence before fellowship	Tel-Aviv and Central Israel	196	66.0
	Jerusalem and West Bank	31	10.4
	Haifa and Northern Israel	50	16.8
	Southern Israel	20	6.7
Marital Status	Married or known in public	284	95.6
	Divorced	4	1.3
	Single	9	3.0
Number of children—Mean (IQR)		2.4	(2–3)
Oldest Child Age- Mean (IQR)		7.8	(5–10)
Youngest Child Age—Mean (IQR)		3.3	(2–5)
Religiousness*	Secular	204	68.7
	Religious	91	30.6
Country of Medical School	Israel	225	75.8
	Abroad	56	18.9
Location of origin hospital**	Central Israel	263	88.6
	Periphery	34	11.4
Country of fellowship	USA	134	45.1
	Canada	89	30.0
	UK	22	7.4
	Australia and New-Zealand	31	10.4
	Europe	15	5.1
	Other	6	2.0
Type of fellowship	Clinical	189	63.6
	Research	32	10.8
	Combined- clinical and research	76	25.6
Medical Specialty***	Internal Medicine, Pediatrics, Oncology Anesthesia, and Other Non-surgical	123	41%
	General Surgery, Neurosurgery, Cardiothoracic surgery	40	13%
	Orthopedic Surgery, Urology, ENT, Ophthalmology, Plastic surgery, Maxillofacial Surgery	88	29%
	Obstetrics and Gynecology	24	8%
	Psychiatry	5	2%
	Radiology	17	6%

There are a few missing values due to participants who chose not to answer specific questions, the relative proportion (%) accounts for missing values

*ENT* Ear nose and throat surgery, *IQR* Interquartile range, *N* number, *UK* United Kingdom, *USA* United States of America

^*^Religiousness was assessed on a scale of 1 to 5 and answers ≥ 1 were regarded as religious

^**^Location of origin hospital – periphery was defined as hospitals entitled to a periphery grant

^***^Detailed list of medical Specialties in Table S1

Of those currently residing abroad, 48 (31%) have already completed their fellowship, with an average time since completion of 3.3 years.

### Perceived benefits and difficulties of the fellowship, and support from the Israeli health system

Respondents rated the benefits of their fellowships as 4.6 (SD 0.7) on a scale of 1 to 5. Overall 72% reported that there is no accredited fellowship in their niche in Israel, and 54% stated that there is no fellowship accredited or non-accredited, in their specific subspeciality in Israel. When asked about the three primary motivations for pursuing an international fellowship, 73% cited broader clinical exposure compared to Israel, 47% valued exposure to different healthcare systems, and 46% sought an adventure (i.e. personal and family experiences abroad). Only 6.4% indicated a desire to immigrate from Israel to another country as a primary motivation. Before departure, the average confidence level in returning to Israel was 4.2 ± 1.0 on a 1 to 5 scale. Only 25.8% embarked with low to medium confidence (confidence ≤ 3).

We assessed the support from the Israeli health system when embarking on a fellowship in a few aspects where responders stated they had significant difficulties. The complex application process poses a substantial obstacle for fellows, and 47% mentioned this was a major difficulty in the process, 66% mentioned they received some form of assistance in the application process, mostly a connection with a fellowship program through an Israeli attending (40%) or a prior Israeli fellow in the same program (38%). Only 2% participated in a fellowship with a formal agreement through a collaboration between an Israeli institution and a fellowship program abroad.

The financial burden of the fellowship is another major difficulty stated by survey responders (40% rated this as a major difficulty) and the average direct personal expenses from savings and loans associated with the fellowship were 35,546 USD (SD 40,693), excluding indirect income loss, such as reduced income during the fellowship and reduced contributions to savings accounts and pensions. Overall, 40% (n = 120) received financial assistance from an Israeli fund, either their employer or an external grant. For those who received financial assistance the average amount covered was 10% (SD 18) of the overall expenses of the fellowship.

Another major difficulty stated by 31% of responders was occupational uncertainty. Only 55.3% had a secured job in a hospital that was promised before embarking on fellowship, for 97% of them this was the same workplace where they had been working before their fellowship.

Out of those who did not receive a secured job offer before their fellowship, only 17% stated that there is an ample number of doctors in their specialty in Israeli hospitals, and 12% stated that there was an ample number of doctors in Israel in their specialty both in hospitals and community settings.

### Push and pull factors

A majority of the survey respondents (72%) envision their future within the next decade in Israel, however, among those currently residing abroad this proportion is lower 88/156 (56%).

**Fig. 2:**
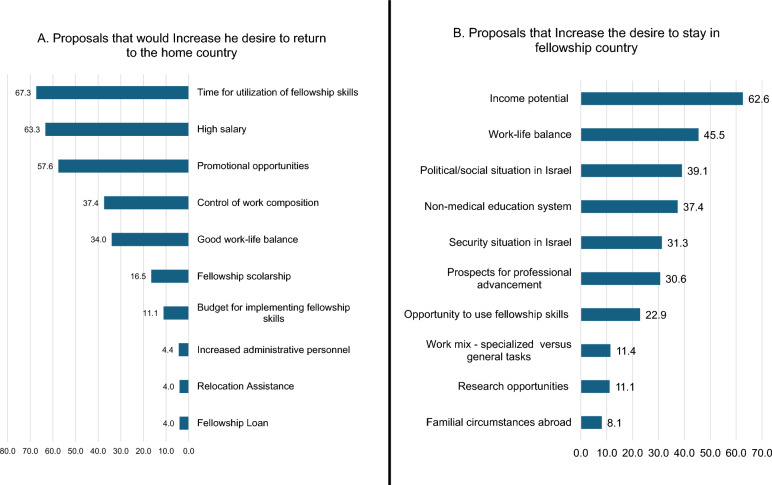
Push and pull factors: **A** Push factors increasing the desire to return to Israel- the proportion of responders that rated each option in the top three places regarding their impact on the desire to **return to Israel** at the end of the fellowship period—Assistance with relocation (language, family and bureaucracy.), Fellowship loan, A scholarship for the fellowship, Adding personnel to reduce non-clinical workload, Good work-life balance, High salary, Promotional opportunities, Budget for implementing the skills acquired, Ability to control your work structure, A position that utilizes the unique skills acquired during the fellowship”. **B** Pull factors- regarding the desire to **stay in the fellowship country**: Familial circumstances abroad, Political/social situation in Israel, Security situation in Israel, Work-life balance, Income potential, Prospects for professional advancement, Non-medical education- for children, Work-mix specialized versus general tasks, protected research time, Research budget and resources. A job that showcases the skills acquired during fellowship

Respondents rated ten factors influencing their desire to stay in the host country (push factors) or return to the origin country (pull-back factors). We summed the number of participants that rated each factor in the top three places. The top push factors were financial opportunities and higher salaries in the fellowship country (59%), the political and social climate in their origin country (49%), and improved work-life balance abroad (39%). Conversely, the top pull-back factors that were stated as those that could encourage a return to Israel included opportunities to utilize skills acquired during the fellowship (67.3%), a high salary upon return to Israel (63.3%), and promotional opportunities (57.6%) as depicted in Fig. 2.

### Demographic characteristics correlated with intention to return

Among the 156 Israeli fellows still abroad, 107 (69%) reported medium to low confidence in returning. Those with lower confidence in returning tended to have fewer children (2.14 ± 1.05 vs. 2.67 ± 1.37, *p* = 0.002) and younger children (the age of the oldest child −6.91 ± 2.88 vs. 8.4 ± 3.5, *p* = 0.022). A higher percentage of secular individuals was found among those with low confidence in returning (77.9% vs 56.5% *p* = 0.011) (Supplementary Table S2). Additionally, confidence in returning to Israel decreased as the length of stay abroad increased. Among those who had already completed their fellowship and still resided abroad, 92% reported medium–low confidence in returning. In contrast, of the 69 participants who were in their first year of fellowship, 58% reported medium–low confidence in returning.

### Intervention factors and confidence in return

**Fig. 3:**
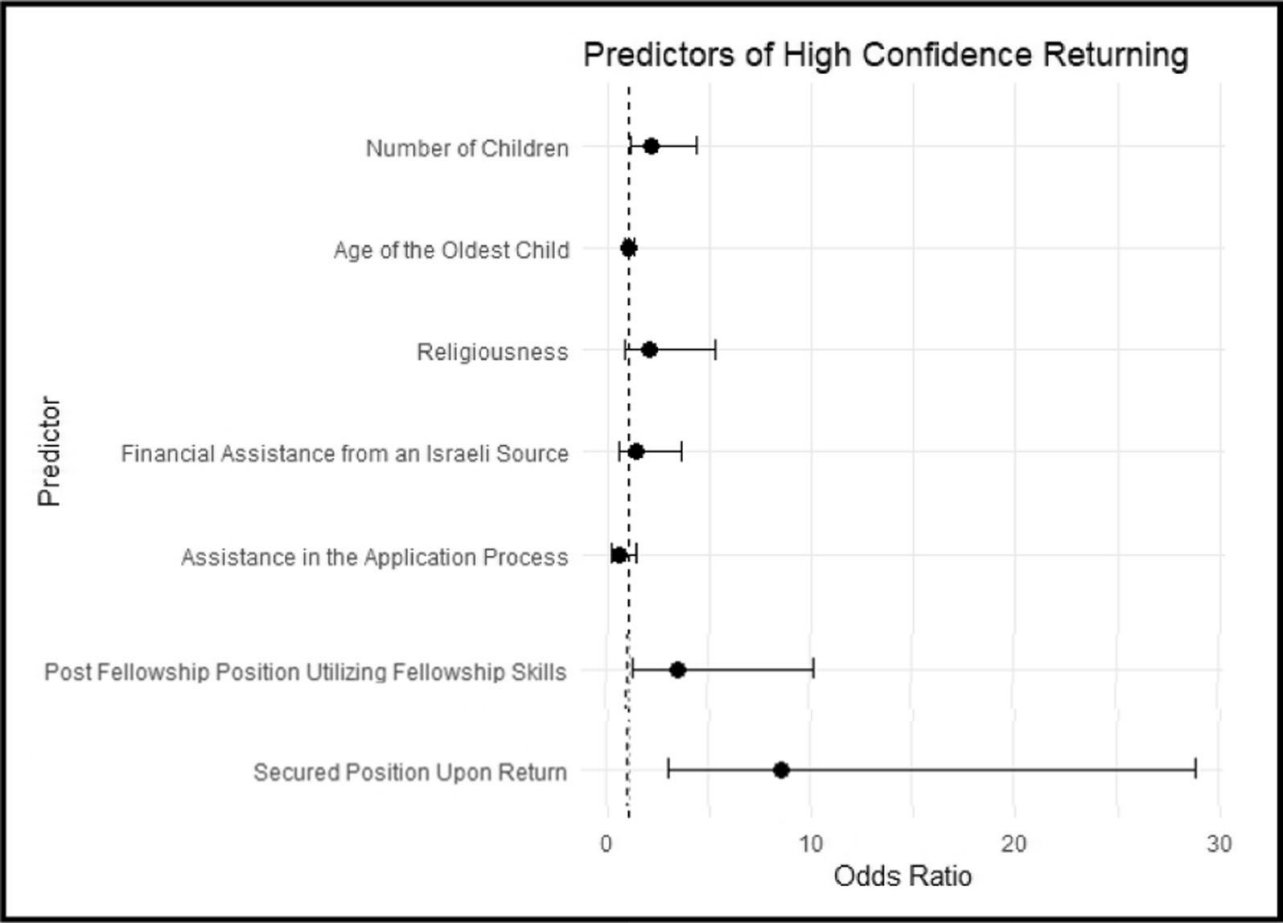
Predictors of high confidence returning to the home country Among Israeli Fellows Abroad: A Logistic Regression Analysis

We examined how support from the Israeli health system affected the confidence of returning responders living abroad. These factors included receiving a scholarship from an Israeli hospital before the fellowship, assistance from Israeli colleagues and superiors during the application process, a guaranteed job opportunity post-fellowship, and an opportunity for a post-fellowship position that would utilize the fellowship skills. We ran a multivariable logistic regression analysis for the 156 participants that included both demographic characteristics found to be significant in a univariant analysis (number of children, age of the oldest child, and religiousness), as well as the intervention factors- we found that a guaranteed job opportunity post-fellowship had an odds ratio (OR) for high confidence in returning to Israel of 8.6 (95% CI 3.1–28.9) and that an opportunity for a post-fellowship position that would utilize the fellowship skills had an OR of a 3.5 (95% CI 1.3–10.2) (Fig. 3, Supplementary Table S3). In a sensitivity analysis of 121 participants, we found similar results after excluding participants who mentioned that they had already secured a position abroad (Supplementary Table S4).

We found that respondents were generally open to various occupational opportunities, including those in Israel's periphery, which typically experiences a severe shortage of medical professionals. Of the 116 respondents who answered the question about their intended geographical region of work upon return, 30 (26%) indicated plans to work in the northern or southern regions of Israel. Notably, only 20 (17%) of these 116 had lived in these areas before starting their fellowship.

### Effects of geopolitical turmoil on Israeli fellows abroad

Since our study was conducted during a time of geopolitical turmoil in Israel and among Jewish communities abroad, we asked specifically how each one of the following had affected the decision to return from fellowship, among participants residing abroad: The October 7th war, the political situation in Israel, and increased antisemitic events and anti-Israel protests abroad. The October 7th war had a mixed effect, decreasing the pull-back motivation among 52%, however, increasing the motivation to return among other 30% of fellows. The political situation decreased the motivation to return to Israel among 76% of responders and increased the motivation among 7% only. Moreover, the antisemitic events decreased the motivation to return for 7% and increased the motivation among 40% (Supplementary Fig. S1).

## Discussion

In this study, we surveyed Israeli physicians either undergoing or have completed post-residency fellowship training within the last decade. Our study characterized this cohort, examining the challenges they encounter and the support systems available to them, while also attempting to discern the factors that influence their decision to return to their native country. We categorized these influencing factors into three levels: micro-factors, which include personal family circumstances, where the number and ages of children, as well as religiousness, have been significant; meso-factors, which pertain to professional status—including career opportunities to apply skills learned during the fellowship, prospects for future advancement, and financial compensation; and macro-factors, which are geopolitical and have become increasingly significant amid the current reality in Israel.

The shortage of healthcare workers is a global challenge, accelerated during coronavirus disease 2019 (COVID-19), leading to increased concern and strategy development in many countries, for retaining healthcare workers [1].

Israel's healthcare system is internationally recognized for its innovation, efficiency, and high performance. Despite having lower health expenditures compared to other high-income countries, Israel demonstrates high life expectancy and low rates of maternal and infant mortality compared to other countries in Europe [17]. However, Israel is also dealing with a significant "brain drain”, a trend exacerbated by doctors' increasing reluctance to return to the country after completing their training abroad. In May 2023, The Organization for Economic Cooperation and Development (OECD) reported that Israel has an average of 3.7 doctors per 1000 population, falling below the OECD average [18]. These numbers represent all licensed doctors, encompassing those in managerial, educational, research, and other roles (constituting an additional 5–10% of doctors) as well as those who have transitioned out of the health sector and those who have emigrated, leading to a substantial overestimation of the number of actively practicing doctors. Additionally, nearly half of all doctors in Israel are aged 55 and over [18]. Despite a significant increase in the number of medical students and new residents over the past decade, Israel still lags behind most OECD countries in terms of medical graduates [18].

Our survey reveals that the majority of doctors who are abroad for their fellowship training have low confidence in returning to Israel and that nearly half do not see their future in the following decade in Israel, despite the very high confidence in returning that they felt when embarking on fellowship, and that the vast majority of them did not initially intend to immigrate.

We lack historical data to compare the current situation with the past. However, our survey indicates that current Israeli realities significantly influence the decision to return. While this journal focuses on health policy, it is important to recognize that factors beyond the health system and policy also play a role in encouraging fellows to remain abroad. Therefore, addressing the factors within the control of health policymakers becomes increasingly urgent and crucial.

### Implications on policymaking

Our research indicates that the decision to return from the fellowship presents several challenges that could be mitigated through Israeli Health system policy changes. When designing an intervention program to enhance the motivation of Israeli physicians to return to Israel after their fellowships, it is crucial to ensure flexibility to accommodate changing life circumstances while preserving occupational freedom, freedom of movement, and privacy. Despite these constraints, practical solutions can be advanced. We propose applying these measures:

- **Mapping of system needs**- Most fellows who did not receive job offers before starting their fellowship believe that their specialty is in shortage in Israel (88%), though this is a subjective opinion. Currently, there is no accessible database for physicians, or even medical students, to compare the number of specialists in each field with the anticipated need, forcing them to rely on word of mouth and estimations for career decisions. Increasing transparency during the specialty selection process by providing clear databases on the number of existing specialists and the future demand for specialists, including breakdowns by specialty and work setting (hospital versus community), can be beneficial. Moreover, since the cohort undertaking fellowships represents a small fraction of physicians and most are expected to return to hospitals, a more granular mapping of system needs is essential. This mapping should include specific regional and hospital requirements for specialists, based on factors such as population growth, the current age of practicing physicians, and anticipated hospital development.
- **Enhancing mobility between hospitals**- The observation that preparedness to live and work in Israel's periphery increased by 50% upon returning from fellowship underscores the importance of targeted measures and interventions. However, hospital managements often lack information about future fellows from other hospitals and face challenges in planning and securing positions two years in advance. As a result, only two of our 156 participants had secured positions at hospitals other than those they had worked in before their fellowship, with only one in a peripheral region. Establishing a national database of Israeli physicians embarking on fellowships and an inventory of anticipated available positions could enhance the placement process, facilitating placements in hospitals beyond their initial departure sites, especially in peripheral hospitals. Physicians who have relocated their families abroad may find moving within Israel easier, and financial incentives covering fellowship years could further facilitate this process.
- **Advancing early job offers**- A recurring issue mentioned in open-text responses and interviews is the timing of securing positions in Israel. Department heads and management in Israel, often finalize contracts just before a role begins, which poses a challenge for those relocating their families. It is important to allow managerial flexibility for hospital and department heads to offer positions well in advance. The global standard is to sign a contract for a position after a fellowship approximately one year in advance, while in Israel this period is dramatically shorter, pushing physicians toward the foreign offers that allow certainty well in advance.
- **Enhancing application and financial support before and during fellowship**- Even when physicians secure positions upon their return, 98% are not “sent” on their fellowships but are expected to find and apply for them independently. Financial assistance, when available, covers up to 10% of the total expenses. Our in-depth interviews revealed that the discrepancy between the expectation for fellowships to be a required step for those aiming to practice in an academic setting and the reality of them being an entirely independent process with minimal support from Israeli employers is a major source of frustration, contributing to a diminished sense of obligation to return. Conditional grants for fellowship departure should be considered, with a preference for physicians who plan to return to underserved locations or to needed specialties.
- **Financial compensation upon return**- Although many participants recognized the prospect of higher income as a significant factor that could encourage their return to Israel, this aspect was not included in the analysis due to uncertainties surrounding income and employment terms upon return, even for those with a guaranteed job offer. This lack of clarity made it challenging to compare individuals with higher salaries upon return to those without. Nevertheless, we believe that financial compensation is a crucial consideration, as it emerged as a major factor influencing the decision to return, as highlighted not only in the survey itself but also in the preliminary in-depth interviews and free-text responses. Recruitment packages for physicians during fellowships should guarantee a position at a hospital in the specific field with the possibility of full-time work or combined roles involving both hospital and community work. Such positions should offer professional challenges with dedicated time for research and teaching, as well as financial compensation sufficient to support a full-time job without the need for additional income from other roles and should be offered preferably before embarking on a fellowship, strengthening commitment on both sides and reducing career uncertainty.
- **Mentoring**- Mentoring healthcare workers can improve organizational commitment and reduce turnover [1]. Although the population in our study is somewhat different than other studies, we suggest addressing physician shortages and brain drain with mentoring doctors during their early career planning.
- **Assistive personnel**- Employing assistive personnel, such as physician assistant and medical assistant, can alleviate workload and potentially improve physician retention [19, 20].

While financial incentives and rewards are important, a significant part of the solution involves not only financial considerations but also reorganizing the system to provide job security, advance planning of major transitions, and transparency in these processes. These measures do not require substantial budgets and can lead to meaningful improvements.

Several countries have implemented immigration policies and financial incentives to attract and retain physicians, including the UK [11]. Sub-Saharan countries have experimented with financial incentives to mitigate physician emigration, such as compensating for overtime work, subsidizing housing and transportation, and increasing salaries to match inflation. While these programs have shown positive trends, more data is needed to assess their full effectiveness [21].

### Limitations

This study encountered several limitations. Primarily, there is no formal database of all Israelis pursuing fellowships abroad. Furthermore, we lack information regarding the breakdown of the original cohort of Israeli fellows abroad by gender, specialty, and country where the fellowship was conducted. Thus, we cannot ascertain if our survey represents a true cross-section of the population of Israeli fellows. Previous studies have identified discrepancies in compliance with electronic surveys across different specialties [22], and we assume we might encounter a similar bias. Despite these challenges, we achieved the planned response rate, offering insights into trends relevant to the study objectives.

Secondly, a potential limitation is selection bias. Although the survey was distributed through various methods, we lacked information on individuals who received the survey but chose not to participate. This gap could influence the results, especially in understanding the motives of those deciding not to return to Israel. Informal feedback suggested reasons for non-participation include a lack of intention to return to Israel, thus a disinterest in offering insights into the local health system, or a reluctance to confront what they perceive as a poor decision to return.

## Conclusions

In summary, this study highlights the urgent issue of physician brain drain among Israeli physicians returning from fellowship training abroad. Key factors like occupational stability and support from the Israeli health system are crucial for encouraging their return. Immediate, structured interventions are needed to address this challenge and retain our valuable medical professionals.

## Supplementary Information

Additional file 1.

## Data Availability

Data availability is not applicable to this article due to their containing information that could compromise the privacy of research participants.

## Abbreviations

COVID-19: Coronavirus disease 2019
IRB: Institutional Review Board
N: Number
OECD: Organization for Economic Cooperation and Development
SD: Standard deviation
UK: United Kingdom
USA: United States of America
USD: United States Dollar
VS: Versus
