# Supplementary material for: Motivations of Israeli physicians to return, or not, to Israel after their fellowship abroad
Source: Isr J Health Policy Res. 2024 Nov 5;13:65. doi: 10.1186/s13584-024-00652-6 (PMC11539758; doi:10.1186/s13584-024-00652-6)
Supplement: Supplementary file 1 — Additional file 1. [file 13584_2024_652_MOESM1_ESM.docx]

**Supplementary**

Contents

Supplementary Methods S1- Structured Interviews……………………………………………………………….2

[Supplementary Methods S2- Survey of Motivations, Opportunities, and Challenges during the Fellowship Period Abroad for Israeli Physicians and Considerations for Returning to Israel 3](#_Toc176785599)

[Supplementary Table S1- Participants specialties 15](#_Toc176785600)

[Supplementary Table S2- Demographic characteristics correlated with intention to Return among respondents residing abroad. 16](#_Toc176785601)

[Supplementary Table S3- Multivariable logistic regression model predictors for high confidence returning among participants residing abroad 18](#_Toc176785602)

[Supplementary Table S4- Multivariable logistic regression model- sensitivity analysis- predictors for high confidence returning among participants residing abroad with patients with a secured position abroad excluded 18](#_Toc176785603)

[Supplementary Figure S1- Effects of geopolitical turmoil on Israeli fellows abroad. 19](#_Toc176785604)

## Supplementary Methods S1- Structured Interviews

Introduction

This in-depth questionnaire is used to design a survey for Israeli physicians who have completed or are currently undergoing a fellowship abroad. The inclusion criteria are identical to those for the survey.

Presenting the Research to the Participant

"Hi, I and other physicians are conducting a research survey to examine the motivations, opportunities, and challenges faced by Israeli physicians during their fellowships abroad, as well as their considerations for returning to Israel, the reasons why physicians choose to stay abroad or return to Israel, and what could strengthen the desire to return to Israel compared to staying abroad. I have some guiding questions, but feel free to say whatever comes to mind, and I will record the conversation in writing. Your information will remain anonymous."

Guiding Questions

1. What fellowship are you pursuing/completed? Tell us about what you do, and why it is important.
2. How will your workload be like, comparing between your fellowship time abroad versus your work in Israel?
3. Why did you pursue a fellowship?
4. How did you find the fellowship you pursued?
5. What changes do you think will happen in your life after returning to Israel following the fellowship?
6. How do you think the fellowship will contribute to healthcare in Israel in the future?
7. How did you manage financially during the fellowship?
8. What are your plans for returning to Israel? How definite is this, and how committed do you feel to returning?
9. If you had decided to stay in the country where you did your fellowship, how realistic would it have been, and what challenges would you have faced?
10. Did you consider staying in the country where you did your fellowship? What were the main reasons for considering this?
11. What changes in the healthcare system or in Israel would positively influence your motivation to return to Israel?
12. If you were in a decision-making position in a large and influential body such as the Ministry of Treasury, Ministry of Health, hospital, medical association, Ministry of Immigration, Social Security, or Tax Authority, what ideas or tools would you suggest to strengthen the desire of Israeli physicians abroad who went for fellowships to return to Israel at the end of the fellowship?
13. If you had implemented the suggestion you made in the previous question, how much would it have affected your motivation to return to Israel after the fellowship (if you have already returned, how satisfied would you feel with returning to Israel if you had implemented what you suggested)?
14. What are the considerations for staying or returning, personally, professionally, and in terms of the country's situation? Which of these considerations is most important to you, and has this changed between the time you left for the fellowship, the time you were supposed to decide to return, and now if you have already returned?
15. Is there a difference between men and women in terms of fellowships? What do you think the difference is?
16. Do you feel that you "represent" the other people who are in the fellowship? Do you think your case is different or exceptional?
17. If you stayed in the country where you did your fellowship, how much of your work would require low/medium/high level of training? If you return to Israel, how do you see this division? If there is a gap between the countries, does this gap bother you?
18. Is there anything else you would like to say?

## Supplementary Methods S2- Survey of Motivations, Opportunities, and Challenges during the Fellowship Period Abroad for Israeli Physicians and Considerations for Returning to Israel

The following survey is entirely anonymous.

No personally identifiable information, such as email addresses or phone numbers, will be collected.

- The survey length is about 15-20 minutes.

- Please answer only once and be as accurate as possible.

- If you have done multiple fellowships, please consider your most recent one.

- If you belong to a group of physicians in a partnership with physicians in fellowship* - only one of the partners will participate in this survey.

# Be sure to complete the survey and press "Submit" to save the survey results #

- The purpose of the survey is to characterize Israeli physicians' experiences, challenges, motivations, and opportunities during or after their fellowship abroad.

- Filling out the survey and responding to the questionnaire constitutes informed consent to participate anonymously in clinical research whose results will be published in the medical and non-medical press.

The results will be distributed to policy-makers in the State of Israel and may contribute to improving planning and support provided to physicians before, during, and after the fellowship.

The principal investigators, Dr. Lior Seluk and Dr. Maayan Gilboa are Israeli physicians currently pursuing fellowships in the USA.

For details about the survey and questions, please contact us via email:

[mayangilboa@yahoo.com](mailto:mayangilboa@yahoo.com) , [liorseluk@gmail.com](mailto:liorseluk@gmail.com)

*Fellowship - a clinical or research training period in one of the medical fields.

I have trained and obtained a medical license in Israel. I agree to fill out the survey anonymously and that the information provided will be used for the abovementioned purposes.

- Yes

- No      (:"Since you indicated 'No' in the previous question, you are not eligible to complete the survey. Thank you for your time and cooperation. Goodbye.")

1. Please select your gender (self-defined)

   - Male

   - Female

- Other:

2. Year of Birth (YYYY)

3. Please indicate the year of completion of your last residency (primary or secondary) in Israel. (YYYY)

4. Please indicate the year in which you started your fellowship (YYYY)

5. Indicate the total number of fellowships planned or completed.

6. What is the fellowship's total duration (in months)? (*Sum up the duration if more than one fellowship was planned/performed)

7. Marital status at the time of leaving for the fellowship.

   - Married or partner

   - Divorced (children in my custody)

   - Divorced (children not in my custody)

   - Single

   - Widowed

   - Other

8. Indicate the number of children you had when embarking on your fellowship.

9. What was the age of the oldest child when you embarked on your fellowship (leave blank if there were no children)?

10. What was the age of the youngest child at the time of embarking on your fellowship (leave blank if no children)?

11. Indicate the number of children born during the fellowship.

12. Country of birth

   - Israel

   - Other

13. Ethnic origin

   - Jewish

   - Muslim

   - Christian

   - Druze

   - Other / Prefer not to answer

14. How would you define yourself religiously?

   1-5 (1 – Secular, 5 - Ultra-Orthodox)

15. Place of residence at the time of filling out the survey

   - Israel

   - Fellowship country

   - Another country

16. Residential district in Israel **before** traveling for the fellowship.

   - Northern district

   - Haifa

   - Sharon

   - Tel Aviv and Gush Dan

   - Shfela

   - Jerusalem

   - Judea and Samaria

   - Southern district

17. What was the classification of the residential area where you lived in Israel before leaving for the fellowship?

   - Urban

   - Rural (Kibbutz, Moshav, Kfar, etc.)

   - Other

18. Do you plan to live in Israel in the next decade?

   - Yes

   - No

19. Where did you study medicine (in which country)?

20. What is your primary field of specialization?

   - Oncology

   - Public Health

   - Geriatrics

   - Anesthesia

   - Obstetrics and Gynecology

   - Urologic Surgery

   - Orthopedic Surgery

   - General Surgery

   - Oral and Maxillofacial Surgery

   - Thoracic Surgery

   - Vascular Surgery

   - Cardiac Surgery

   - ENT and Head & Neck Surgery

   - Dermatology and Venereology

   - Ophthalmology

   - Clinical Microbiology and Clinical Laboratory Sciences

   - Neurosurgery

   - Neurology

   - Psychiatry

   - Pathology

   - Radiology

   - Nuclear Medicine

   - Emergency Medicine

   - Legal Medicine

   - Physical Medicine and Rehabilitation

   - Internal Medicine

   - Occupational Medicine

   - Family Medicine

   - Pediatrics

21. If you underwent a secondary sub-specialization in Israel (e.g., Hand Surgery, Interventional Radiology, Gastroenterology, etc.), what is your area of secondary specialization?

22. Please define your primary workplace before the fellowship.

   - Large hospital

   - Medium-small hospital

   - Psychiatric/Geriatric/Rehabilitation hospital

   - Community

   - Ministry of Health

   - IDF (Israel Defense Forces)

   - Other

23. Was your primary workplace before leaving for the fellowship defined as peripheral by the Ministry of Health? (e.g., Ziv, Nahariya, HaEmek, Shaar Menashe, Lev HaSharon, Ashkelon, Soroka, Yoseftal)

   - Yes

   - No

24. Please order the following proposals according to their impact on your desire to return to Israel at the end of the fellowship period (if they were offered to you).

   *The top proposal- with the most impact, the bottom proposal- with the most negligible impact

   - A scholarship upon leaving for the fellowship

   - High salary upon return to Israel

   - A loan for the fellowship under special conditions

   - A position offering promotional opportunities for professional advancement or a leadership role upon returning to Israel.

   - A position that utilizes the unique skills acquired during the fellowship

   - Ability to control your work structure (specialized clinical work versus general departmental tasks, protected time for research, etc.)

   - Budget for implementing the skills acquired (e.g., medical devices, research equipment, research assistants, support in grant writing, etc.)

- Assistance with relocation (tax and customs wavers, help with children's educational or language gaps, job-finding assistance for a spouse, etc.)

- A position that allows a good work-life balance

- Adding administrative and para-medical personnel to reduce non-clinical workload

25. Is there another proposal that would encourage you or others in your situation to return to Israel?

26. Upon embarking on the fellowship, what was your confidence level that you would return to Israel after the fellowship?

   1-5 (1=I did not intend to return. 5=Very confident I will return to Israel.)

27. Currently, what is your confidence level that you will return to Israel? If you have already returned to Israel, what is your level of satisfaction with your return?

   1-5 (1=I have no intention to return. 5=Very confident in returning to Israel.)

28. What is the level of physician shortage in your field of specialization in hospitals in Israel?

   1-5 (1=Severe shortage. 5=Ample number of doctors.)

29. What is the physician shortage level in your specialization in outpatient networks in Israel?

   1-5 (1=Severe shortage. 5=Ample number of doctors.)

30. How would you assess the professional benefit you gained during the fellowship?

   1-5 (1=I did not gain any benefit. 5=Gain significant benefit.)

31. To what extent do you feel you benefited from the fellowship in each of the following aspects?

   0 Not relevant

   1 Did not gain benefit.

   2 Gained moderate benefit.

   3 Gained significant benefit.

   - The breadth of clinical/surgical exposure during the fellowship

   - Acquisition of research/academic knowledge and skills

   - Acquisition of novel technological knowledge and skills

   - Acquisition of collegial network or reputation for international collaborations

   - Advancement of a specialized medical niche

   - Acquisition of knowledge in medical management

   - Acquisition of knowledge in laboratory management

32. To what extent do you estimate that you will be able to direct the professional benefit you gained during the fellowship towards **improving public medicine in Israel**?

   1-5 (1= Minimal extent. 5= To a great extent.)

33. What were your sources of income (as an individual and as a family) during the fellowship?

   *Please mark all that are relevant to you

   - Salary from the fellowship institution

   - Grant or scholarship from a hospital in Israel, network (Kupat Holim), or Ministry of Health

   - Grant or scholarship from a different organization in Israel (Professional association, Israeli Medical Association, Cancer Association, etc.)

   - Additional remote work you do during your fellowship

   - Spouse work remotely from Israel

   - Spouse work in the fellowship country

   - Rental income or any other passive income from properties/companies in Israel

   - Financial support from immediate family

   - Use of own savings/loans

   - Other

34. Please estimate what percentage of your expenses during the fellowship was covered by your fellowship salary.

35. Please estimate what percentage of your expenses during the fellowship were covered by grants received in Israel.

   *This question is separate from question 34. There is no need to sum up questions 34 and 35 to 100%.

36. Please estimate the amount (in new Israeli shekels) you spent from your savings for the fellowship.

   *If you did not use your personal savings, please write "0."

37. Did you receive financial support for your fellowship from any organization in Israel?

   - Yes

   - No

38. Did you formally commit (with a signed contract) to return to the same workplace after the fellowship ends?

   - Yes

   - No

   - Not relevant

39. please specify the type of your fellowship

   - Clinical

   - Research

   - Combined

   - Other

40. Fellowship country?

   - USA

   - Canada

   - Australia

   - UK (United Kingdom)

   - Western Europe (Germany, France, Spain, etc.)

   - South and Central America

   - East Asia (China, India, South Korea, etc.)

   - Other

41. Is the fellowship officially accredited?

   - Yes

   - No

42. What is your fellowship's level of hands-on training (surgeries, endoscopies, catheterizations, etc.)?

   1-5 (1=No hands-on training at all. 5=Exclusively hands-on fellowship.)

43. What exams were you required to fulfill to be accepted for fellowship?

   - Licensing exam in medicine in the destination country (e.g., USMLE)

   - Language exam

   - No exams were necessary

   - Other

44. What are the main reasons for pursuing a fellowship abroad?

*You can select up to 3 answers

   - Pressured by the workplace in Israel and/necessary for advancement in Israel

   - Broader clinical exposure in comparison to Israel

   - Significant research opportunities in comparison to Israel

   - Exposure to different healthcare systems, broadening horizons

   - Family circumstances (fellowship or relocation of another family member)

   - Desire to immigrate to another country

   - Seeking an adventure

   - Other

45. Does the fellowship exist in Israel in any form?

   - No

   - Yes, as a non-certified program

   - Yes, as a certified program

46. How did you find and secure your fellowship position?

*You may select more than one

   - Through an Israeli doctor who has done or is doing the fellowship in the same program

   - Recommendations about the program from other Israeli doctors

   - Personal acquaintance of senior physicians in Israel with senior physicians from the institution where the fellowship is conducted

   - Collaboration between an Israeli institution and the fellowship program abroad

   - Contacts I made myself (at a conference, social network, by email, etc.)

   - Independent search for a program that interests me

   - Registration for a fellowship without prior acquaintance (e.g., through MATCH)

   - Other

47. How many work hours per week (on average) are you required to work during the fellowship?

48. Did you travel to the fellowship with a spouse?

   - Yes

   - No

   - Not relevant

49. Is your spouse a physician?

   - Yes

   - No

   - Not relevant

50. Did your spouse do a fellowship concurrently?

   - Yes, and the fellowship was more demanding than mine

   - Yes, and the fellowship was as equally demanding as mine

   - Yes, and the fellowship was less demanding than mine

   - No

   - Not relevant

51. Did you travel to the fellowship with your children?

   - Yes

   - No

   - Not relevant

52. During the fellowship, was your spouse able to work?

   - Yes

   - No

   - Not relevant

53. If your spouse did not work, what was the reason for not working?

   - It was not possible for him/her to work due to family or personal reasons

   - It was not possible due to legal/bureaucratic restrictions

   - He/She was not interested in working during the fellowship

   - Other

54. Many challenges characterize the fellowship period. Please indicate the level of challenge each of the following posed for you:

   0 Not relevant

   1 Did not pose a challenge.

   2 Moderate challenges.

   3 Significant challenges.

   - Licensing exams and language tests

   - Finding a program and application submission

   - Interview and acceptance process

   - Personal/family financial status

   - Ability to speak the local language

   - Cultural differences

   - Social/emotional difficulties of the applicant

   - Differences in organizational/work culture

   - Gaps in professional skills

   - Work-life balance

   - Occupational difficulties of the spouse

   - Social/emotional difficulties of the spouse

   - Academic difficulties of the children

   - Social/emotional difficulties of the children

   - Geographic distance from family in Israel

   - Uncertainty about your future employment

55. Which of the following statements best describes your spouse's employment status during the fellowship?

   - It was a career opportunity for him/her

   - There was no change in his/her career during the fellowship

   - He/She had to compromise professionally or in terms of earning ability during the fellowship

   - The fellowship harmed the career progression of the spouse

   - Not relevant

56. The fellowship period can be difficult for the applicant and his/her spouse/family. How would you rate the difficulty of your fellowship experience?

   1-5 (1= Not significant. 5= Very significant.)

57. Please describe if there were other significant challenges worth mentioning.

58. Before traveling for the fellowship, what was the level of support you received from your spouse for going?

   1-5 (1= Not at all. 5= Very much.)

59. After adjusting to the fellowship, what level of support did your spouse provide you with regarding it?

   1-5 (1= Not at all. 5= Very much.)

60. To what extent did your children adapt (academically, socially, and linguistically) during the fellowship?

   1-5 (1= Did not adapt. 5= Adapted very well.)

61. How difficult would it be to find a job in your field in the fellowship country?

   1-5 (1= Very easy. 5= Very difficult.)

62. Please order the following characteristics of your life in the fellowship country according to their impact on your desire to stay in the fellowship country.

   *The highest feature - most influential. The lowest - least influential or not influential at all

   - Earning potential

   - Prospects for professional advancement/ leadership role

   - Work-life balance

   - Security status in Israel

   - Political/social situation in Israel

   - Education for children in the fellowship country

   - Familial circumstances abroad

   - A job that showcases the skills acquired during fellowship

   - Research opportunities and academic advancement (laboratory, laboratory equipment, budgets, research team, etc.)

   - Work mix - specialized clinical tasks versus general departmental tasks, protected time for research

63. To what extent did the following events influence your desire to return to Israel after the fellowship?

If you have already returned, please indicate how much the events influenced your satisfaction with the decision to return

   1 Increased desire to NOT return

   2 Did not influence.

   3 Increased desire to return

   - The judicial reform and protests against the governmental overturn

   - The October 7^th^ attack and the Iron Swords War

   - The rise of antisemitism globally and antisemitic expressions in universities

64. Were you in Israel during the war?

   - Yes, the fellowship ended, and I returned to Israel/living in Israel

   - Yes, I returned for reserve duty

   - Yes, I returned to assist in a civilian medical institution

   - Yes, I volunteered in a civilian capacity

   - No, but my spouse returned

   - No

   - Other

65. What best describes your employment situation upon your return to Israel? If you have already returned to the country – what was your situation on the eve of your return?

- According to the agreement before the fellowship, a job was waiting for me at the workplace I left.

   - According to the agreement before the fellowship, a job was waiting for me at a different workplace from the one I left.

   - While I was abroad, I found a new workplace in Israel (waiting for my return)

   - No job was waiting for me in Israel. It may be difficult to find a job in my field in Israel

   - No job was waiting for me, but finding a job in Israel will not be a problem

   - I do not plan to return to Israel at this stage

   - Other

66. After returning from the fellowship, what type of practice is your principal place of employment expected to be? If you have already returned, where is your main place of employment?

   - Large hospital

   - Medium/small hospital

   - Psychiatric/geriatric/rehabilitation hospital

   - Community

   - Ministry of Health

   - University/research institute

   - Private practice

   - Other

67. Upon your return from the fellowship, in which geographical area do you plan to work?

   - Central

   - Jerusalem

   - Periphery

   - Not returning

68. If you return to Israel, to what extent is the fellowship expected to bring changes? Please respond for each of the following areas.

 If you have already returned to Israel – please indicate what has changed as a result of your return:

   1 Deterioration in situation

   2 No change

   3 Improvement in the situation

   - Salary at a medical institution

   - Prestige among colleagues

   - Significant academic publications

   - Professional advancement/leadership role

   - Income from private practice

   - Opportunity to establish a laboratory or conduct independent research

   - Para-medical work (e.g., high-tech)

   - Prestige among patients

   - Professional satisfaction

   - Family cohesion

   - Engagement in a field of interest

69. Given that the fellowship is intended to develop your profession to the professional/academic edge, what work mix will allow you to engage in your edge specialty (compared to the more basic specialty) after the fellowship?

   1 Will not engage in the field.

   2 Little of the time

   3 Much of the time

   - Upon returning to Israel after the fellowship

   - If I stay to work in the field in another country

70. What is your overall level of satisfaction with the fellowship?

   1-5 (1=Great disappointment. 5=Great satisfaction.)

71. To what extent would you recommend other physicians in a similar situation to yours (family, economic, specialization, immigration status) to undergo the same fellowship experience?

   1-5 (1=Not at all. 5=Strongly recommend.)

72. This is the end of the survey. If you have any additional comments that you think are important to convey to the survey editors or policymakers, please write them here:

You have completed the survey

## Supplementary Table S1- Participants specialties

|  | N | % |
| --- | --- | --- |
| Internal Medicine | 62 | 20.9 |
| Orthopedic Surgery | 35 | 11.8 |
| General Surgery | 29 | 9.8 |
| Pediatrics | 30 | 10.1 |
| Ophthalmology | 23 | 7.7 |
| Obstetrics and Gynecology | 24 | 8.1 |
| Radiology | 17 | 5.7 |
| Anesthesia | 6 | 2.0 |
| Psychiatry | 5 | 1.7 |
| Oncology | 13 | 4.4 |
| Urology | 12 | 4.0 |
| ENT and maxillofacial | 12 | 4.0 |
| Neurosurgery | 8 | 2.7 |
| Cardio-Thoracic | 3 | 1.0 |
| Plastic Surgery | 6 | 2.0 |
| Other | 12 | 4.0 |

## Supplementary Table S2- Demographic characteristics correlated with intention to Return among respondents residing abroad.

|  | | |  |
| --- | --- | --- | --- |
|  | Low confidence returning | High confidence returning | p -value |
| n (%) | 107 (69%) | 49(31%) |  |
| Male | 63 (58.9%) | 30 (62.5%) | 0.725 |
| Avg. Age (SD) | 38.25 (2.6) | 39.96 (2.95) | 0.265 |
| Birth country abroad (%) | 14 (13%) | 9 (18.8) | 0.275 |
| Marital status (%) |  |  | 0.131 |
| Married | 103 (95%) | 43 (90%) |  |
| Single | 4 (3.7%) | 3 (6.3%) |  |
| Divorced with custody | 1 (0.9%) | 0 |  |
| Divorced w/o custody | 0 | 2 (4.2%) |  |
| Accompanied by spouse (%) | 104 (96%) | 40 (85.1%) | 0.019 |
| Avg. Number of children (SD) | 2.14 (1.05) | 2.67 (1.37) | 0.002 |
| Accompanied by children(%) | 100 (100%) | 40 (93%) | 0.026 |
| Avg. age of oldest child (SD) | 6.91 (2.88) | 8.4 (3.51) | 0.022 |
| Avg. age of youngest child (SD) | 3.08 (2.1) | 3.2 (2.27) | 0.818 |
| Non-Jewish ethnicity, n(%) | 11 (10.3%) | 1 (2.1%) | 0.067 |
| Religious | 25 (23.1%) | 20 (5%) | 0.011 |
| Pre-fellowship domicile (%) |  |  | 0.688 |
| Central Israel | 79 (73.1%) | 31 (64.6%) |  |
| Jerusalem | 6 (5.6%) | 4 (8%) |  |
| North District | 15 (13.9%) | 8 (16.7%) |  |
| South District | 8 (7%) | 5 (10.4%) |  |
| Peripheral workplace | 10 (9.3%) | 6 (12.5%) | 0.573 |
| International med. Grads. | 17 (17%) | 5 (11.4%) | 0.424 |
| Type of workplace |  |  | 0.433 |
| Large hospital | 73 (67.6%) | 37 (77.1%) |  |
| Smal-medium hospital | 24 (22.2%) | 9 (18.8%) |  |
| Geriatric/rehab/psych | 1 (0.9%) | 1 (2.1%) |  |
| Community | 9 (8.2%) | 1 (2.1%) |  |
| Residency group (%) |  |  | 0.895 |
| Internal/Peds/Onco/Anest/Other | 51 (47.2%) | 23 (47.9%) |  |
| Surgery/Neurosurg./Cardio-thoracic surg. | 6 (5.6%) | 3 (6.3%) |  |
| Ortho/Uro/ENT/Opht/Plastic/Maxillofacial | 32 (29.6%) | 15 (31.3%) |  |
| OB/GYN | 8 (7.4%) | 5 (10.4%) |  |
| Psychiatry | 3 (2.8%) | 0 |  |
| Radiology | 8 (7.4%) | 2 (4.2%) |  |
| Type of fellowship (%) |  |  |  |
| Clinical | 65 (60.2%) | 34 (70.8%) | 0.351 |
| Research | 11 (10.2%) | 5 (10.4%) |  |
| Combined | 32 (29.6%) | 9 (18.8%) |  |
| Country of fellowship (%) |  |  |  |
| USA | 51 (47.2%) | 21 (43.8%) |  |
| Canada | 35 (32.4%) | 16 (33.3%) | 0.884 |
| UK | 5 (4.6%) | 4 (8.3%) |  |
| Australia + NZ | 13 (12%) | 6 (12.5%) |  |
| Europe | 4 (3.7%) | 1 (2.1%) |  |
| Asia | 0 |  |  |

## Supplementary Table S3- Multivariable logistic regression model predictors for high confidence returning among participants residing abroad

| Predictor | Odds Ratio | Lower CI | Upper CI |
| --- | --- | --- | --- |
| (Intercept) | 0.01 | 0.00 | 0.04 |
| Number of Children | 2.08 | 1.06 | 4.32 |
| Oldest Child Age | 1.02 | 0.84 | 1.25 |
| Religiousness | 2.05 | 0.80 | 5.31 |
| Financial Assistance | 1.41 | 0.55 | 3.58 |
| Application Assistance | 0.53 | 0.20 | 1.37 |
| Promised Work | 8.64 | 3.07 | 28.89 |
| Specified Work in Israel | 3.48 | 1.31 | 10.20 |

## Supplementary Table S4- Multivariable logistic regression model- sensitivity analysis- predictors for high confidence returning among participants residing abroad with patients with a secured position abroad excluded

| Predictor | Odds Ratio | Lower CI | Upper CI |
| --- | --- | --- | --- |
| (Intercept) | 0.01 | 0.00 | 0.00 |
| Number of Children | 2.00 | 2.06 | 8.37 |
| Oldest Child Age | 1.03 | 0.87 | 1.30 |
| Religiousness | 1.75 | 1.20 | 7.88 |
| Financial Assistance | 1.37 | 0.74 | 4.80 |
| Application Assistance | 0.53 | 0.11 | 0.74 |
| Promised Work | 4.53 | 6.98 | 70.99 |
| Specified Work in Israel | 3.29 | 4.06 | 31.60 |

## Supplementary Figure S1- Effects of geopolitical turmoil on Israeli fellows abroad.

Figure S1- Effects of geopolitical turmoil on Israeli fellows abroad- Number of participants that stated that each event has strengthened their intention to stay abroad. Return to Israel or did not affect their decision, among participants currently residing abroad.
